# Shared pathobiology identifies AMPK as a therapeutic target for obesity and autosomal dominant polycystic kidney disease

**DOI:** 10.3389/fmolb.2022.962933

**Published:** 2022-08-23

**Authors:** Ioan-Andrei Iliuta, Xuewen Song, Lauren Pickel, Amirreza Haghighi, Ravi Retnakaran, James Scholey, Hoon-Ki Sung, Gregory R. Steinberg, York Pei

**Affiliations:** ^1^ Division of Nephrology, Department of Medicine, University Health Network and University of Toronto, Toronto, ON, Canada; ^2^ Translational Medicine Program, The Hospital for Sick Children, Toronto, ON, Canada; ^3^ Department of Laboratory Medicine and Pathobiology, University of Toronto, Toronto, ON, Canada; ^4^ Division of Genetics, Department of Medicine, Brigham and Women’s Hospital, Harvard Medical School, Boston, MA, United States; ^5^ Lunenfeld Research Institute, Mount Sinai Hospital, Toronto, ON, Canada; ^6^ Department of Medicine, Centre for Metabolism, Obesity, and Diabetes Research, McMaster University, Hamilton, ON, Canada

**Keywords:** autosomal dominant polycystic kidney disease, energy metabolism, obesity, metabolic dysregulation, AMPK

## Abstract

Autosomal dominant polycystic kidney disease (ADPKD) is the most common Mendelian kidney disease, affecting approximately one in 1,000 births and accounting for 5% of end-stage kidney disease in developed countries. The pathophysiology of ADPKD is strongly linked to metabolic dysregulation, which may be secondary to defective polycystin function. Overweight and obesity are highly prevalent in patients with ADPKD and constitute an independent risk factor for progression. Recent studies have highlighted reduced AMP-activated protein kinase (AMPK) activity, increased mammalian target of rapamycin (mTOR) signaling, and mitochondrial dysfunction as shared pathobiology between ADPKD and overweight/obesity. Notably, mTOR and AMPK are two diametrically opposed sensors of energy metabolism that regulate cell growth and proliferation. However, treatment with the current generation of mTOR inhibitors is poorly tolerated due to their toxicity, making clinical translation difficult. By contrast, multiple preclinical and clinical studies have shown that pharmacological activation of AMPK provides a promising approach to treat ADPKD. In this narrative review, we summarize the pleiotropic functions of AMPK as a regulator of cellular proliferation, macromolecule metabolism, and mitochondrial biogenesis, and discuss the potential for pharmacological activation of AMPK to treat ADPKD and obesity-related kidney disease.

## Introduction

Autosomal dominant polycystic kidney disease (ADPKD) is the most common Mendelian kidney disease (lifetime prevalence of at least 1:1,000 births), accounting for almost 5% of all end-stage kidney disease (ESKD) in developed countries (Harris and Torres, 2009; Lanktree et al., 2018). Mutations in *PKD1* (encoding polycystin 1 or PC1) and *PKD2* (encoding polycystin 2 or PC2) account for 70%–85% and 15%–30% of genetically resolved cases, respectively (Rossetti et al., 2007; Barua et al., 2009; Cornec-Le Gall et al., 2013; Heyer et al., 2016; Hwang et al., 2016). ADPKD is characterized by the slow expansion of innumerable cysts in the kidneys with inflammation and fibrosis as defining features associated with advanced kidney failure in a high proportion of affected individuals (Grantham, 2008; Harris and Torres, 2009). Currently, only tolvaptan, an antagonist of vasopressin V2 receptors is approved to treat ADPKD (Irazabal et al., 2011; Torres et al., 2012; Torres et al., 2017). However, tolvaptan is an expensive drug with serious side effects such as significant polyuria, nocturia, and potential liver toxicity (Irazabal et al., 2011). Hence, there is a pressing need for novel interventions that are safe and effective for patients with ADPKD.

The kidneys are among the most metabolically active organs in the body: although they only represent 0.5% of the human body mass, they consume 10% of the body’s oxygen (Nowak and Hopp, 2020). Consequently, the modulation of key metabolic pathways has generated much interest in the field of ADPKD. The pathophysiology of ADPKD was shown to be strongly linked to metabolic dysregulation, which may occur secondary to defective polycystin function (Distefano et al., 2009; Zheng et al., 2009). Metabolic reprogramming, mediated through novel or repurposed drugs, or through dietary changes (e.g., caloric restriction), holds the promise of improving the course of cystic disease (Nowak and Hopp, 2020; Menezes and Germino, 2019; Podrini et al., 2020; Haumann et al., 2020; Pickel et al., 1093; Warner et al., 2016). Recently, overweight and obesity were demonstrated to be independent predictors of ADPKD progression (Nowak et al., 2018; Nowak et al., 2021). This suggests that weight loss could provide a strategy for controlling cyst growth. In this narrative review, we highlight the shared pathobiology between ADPKD and overweight/obesity. In particular, we focus on the interaction between mammalian target of rapamycin (mTOR) and AMP-activated protein kinase (AMPK) signaling, and their biological functions in the kidneys. mTOR and AMPK are two diametrically opposed sensors of energy metabolism that play a key role in regulating cell growth and proliferation (Steinberg and Kemp, 2009). We summarize the current knowledge on the function of AMPK as a regulator of cellular energy metabolism and modulator of tissue inflammation, and the preclinical and clinical evidence supporting pharmacological activation of AMPK to treat ADPKD and improve obesity-related kidney disease.

### Obesity is clinically associated with more severe autosomal dominant polycystic kidney disease

The World Health Organization (WHO) defines overweight and obesity in adults as a body mass index (BMI) ≥25 kg/m^2^ and ≥30 kg/m^2^, respectively. The global prevalence of obesity in adults was 13% in 2016 (15% of women and 11% of men), a threefold increase since 1975 (https://www.who.int/en/news-room/fact-sheets/detail/obesity-and-overweight, updated 9 June 2021). Worryingly, between 1975 and 2016, the global prevalence of obesity in children and adolescents also increased from 0.7 to 5.6% in girls and from 0.9 to 7.8% in boys (Abarca-Gómez et al., 2017). The rate of increase in BMI since 2000 has slowed down in high-income countries, but has accelerated in some regions of the developing world (NCD Risk Factor Collaboration, 2016). Epidemiologic studies have highlighted high BMI as a risk factor for numerous chronic conditions, including cardiovascular disease, diabetes mellitus, chronic kidney disease (CKD), cancer, and musculoskeletal disorders (The GBD 2015 Obesity Collaborators Ashkan Afshin et al., 2017). Obesity results from energy intake exceeding energy expenditure, which leads to adipose tissue expansion (Rosen and Spiegelman, 2006). Contributors to this energy imbalance are numerous, including genetic, epigenetic, physiological, environmental, sociocultural, and behavioral factors (Fall et al., 2017; Gadde et al., 2018).

Although it was noted almost 20 years ago that the number of patients with ADPKD and concomitant obesity has been increasing, only recently has the impact of high BMI on ADPKD been explored (Schrier et al., 2003). Nowak et al. (2018) observed that, in 441 non-diabetic subjects with early-stage ADPKD, more than half of the cohort was overweight or obese. Importantly, BMI was independently associated with a greater annual percent change in total kidney volume (TKV). Obesity, compared to normal weight, was also independently associated with a greater decline in eGFR (Nowak et al., 2018). A later study confirmed some of these observations using patients at high risk of rapid progression from the Tolvaptan Efficacy and Safety in Management of Autosomal Dominant Polycystic Kidney Disease and Its Outcomes (TEMPO) 3:4 trial (Nowak et al., 2021).

Obesity commonly co-occurs with the metabolic syndrome. The latter is characterized by a cluster of cardiometabolic risk factors including abdominal obesity, high blood pressure, elevated fasting blood glucose, and dyslipidemia (i.e., high triglycerides and low HDL cholesterol) (Alberti et al., 2009). Pietrzak-Nowacka et al. (2009) found that hypertension was the main component of the metabolic syndrome to correlate with ADPKD, whereas BMI and waist-to-hip ratio did not differ compared to age- and gender-matched controls. Fasting glucose levels were higher in patients with ADPKD, but the difference in blood glucose did not persist after an oral glucose tolerance test. However, these findings must be interpreted with caution, as BMI was not adjusted for kidney and liver volume. Reed et al. (2012) described larger kidney volumes in patients with ADPKD and type 2 diabetes, compared to those with ADPKD alone. However, since BMI was higher on average in the group with type 2 diabetes, it remains unclear whether it was the dysglycemia or elevated BMI that contributed to the results.

Several signaling pathways relevant to obesity, such as AMPK, are also known to play a major role in the pathobiology of ADPKD. The correlation between BMI and cystic disease progression may be explained by a number of mechanisms including metabolic dysregulation related to nutrient availability, hormonal changes such as hyperinsulinemia, low-grade chronic inflammation, or any combination thereof. Examining the pathways common to both obesity and ADPKD may help generate hypotheses to support future mechanistic studies (Nowak et al., 2018).

### mTORC1 plays a major role in the pathophysiology of autosomal dominant polycystic kidney disease

**FIGURE 1:**
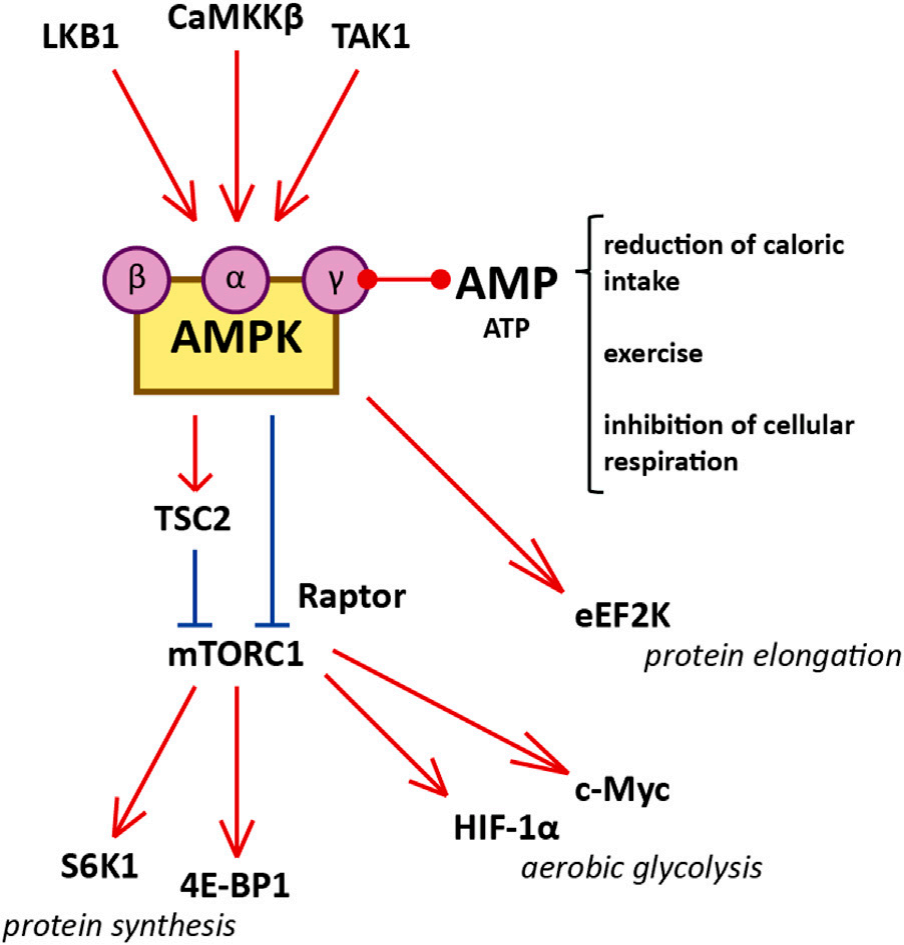
Diagram illustrating the interaction between AMPK and mTORC1, and cellular functions of mTORC1 that influence cystogenesis in ADPKD. Red represents activation and blue represents inhibition. AMPK is a heterotrimer consisting of a catalytic α-subunit, a regulatory β-subunit, and a regulatory γ-subunit assembled in a 1:1:1 ratio. Reduced caloric intake, physical exercise, and interference with electron transport in the mitochondria increase the AMP/ATP ratio. Binding of AMP to the regulatory γ-subunit of AMPK allows the catalytic α-subunit to be phosphorylated at residue Thr172 by one of the three AMPK kinases (LKB1, CaMKKβ, and TAK1). Certain molecules, such as salicylate, can activate AMPK directly by binding the β-subunit. After phosphorylation by the AMPK kinases, AMPK inhibits mTORC1 1) by phosphorylating the Raptor component of the complex (at residues Ser722 and Ser792) and 2) by phosphorylating TSC2 (at residues Thr1227 and Ser1345). The inhibition of mTORC1 suppresses protein synthesis by downregulating S6K1 and 4E-BP1. mTORC1 enhances the translation of c-Myc and HIF-1α, both of which contribute to aerobic glycolysis. Additionally, AMPK inhibits protein translation and elongation by phosphorylating and activating eEF2K.

The mTOR protein is a serine/threonine kinase central to two different complexes: mTOR complex 1 (mTORC1), which includes six protein components, and mTOR complex 2 (mTORC2), which includes seven. mTORC1 responds to available amino acids and oxygen, growth factors, DNA homeostasis, and cellular energy levels to activate cell proliferation by enhancing protein synthesis, stimulating the cell cycle, and inhibiting autophagy. On the other hand, mTORC2 is involved in the organization of the actin cytoskeleton and regulates mTORC1 function (Laplante and Sabatini, 2012; Yoon, 2017; Boutouja et al., 2019). mTORC1 is downregulated by AMPK, which phosphorylates and inactivates the Raptor component of the complex, and phosphorylates and activates tuberous sclerosis complex 2 (TSC2), a negative regulator upstream of mTORC1 (Figure 1). (Boutouja et al., 2019) mTORC1 signaling promotes protein synthesis by phosphorylating the translational regulators S6 kinase 1 (S6K1) and 4E-binding protein 1 (4E-BP1) (Laplante and Sabatini, 2012). Moreover, mTORC1 is a potent suppressor of autophagy *via* phosphorylation and inhibition of unc-51-like kinase 1 (ULK1) (Laplante and Sabatini, 2012). mTORC1 is activated in the kidney proximal tubules of obese mice fed a high-fat diet (HFD), and long-term lipid overload was recently shown to induce lysosomal dysfunction, impaired autophagy, and susceptibility to kidney injury (Yamahara et al., 2013; Yamamoto et al., 2017). Enhanced mTORC1 signaling was repeatedly confirmed in the epithelium of kidney cysts, stimulating cell proliferation and inhibiting autophagy, which are both involved in the pathogenesis of ADPKD; multiple preclinical studies of mTORC1 inhibitors sirolimus (rapamycin) and everolimus showed some benefit in rodent models (Table 1). (Tao et al., 2005; Shillingford et al., 2006; Wahl et al., 2006; Wu et al., 2007; Zafar et al., 2009; Shillingford et al., 2010; Zafar et al., 2010; Song et al., 2020)

**TABLE 1:** *In vitro* and animal studies investigating metabolic agents of interest in the treatment of ADPKD.

Agent	Mechanism of action	Study	Experimental model	Evidence
Sirolimus	mTOR inhibitor	Riwanto et al. (2016)	Human ADPKD cells	↓mRNA expression of glycolytic genes and ↓lactate
		Tao et al. (2005)	Han:SPRD rat	↓cyst burden, ↑kidney function
		Wahl et al. (2006)	Han:SPRD rat	↓cyst burden, ↑kidney function
		Shillingford et al. (2006)	*orpk*-rescue mouse	↓cyst burden
		Zafar et al. (2009)	Han:SPRD rat	↓cyst burden, ↑kidney function
		Zafar et al. (2010)	^a^ *Pkd2* ^*WS25/-*^ mouse	↓cyst burden
		Shillingford et al. (2010)	^a^ *Pkd1* ^*cond/cond*^ *:Nestin* ^*cre*^ mouse	↓cyst burden, ↑kidney function, ↓fibrosis
Everolimus	mTOR inhibitor	Wu et al. (2007)	Han:SPRD rat	↓cyst burden, ↑kidney function
2DG	Glycolysis inhibitor/AMPK agonist	Rowe et al. (2013)	• ^a^ *Pkd1* ^*flox/-*^ *:Ksp-Cre* mouse; • ^a^ *Pkd1* ^V/V^ mouse	↓cyst burden ↓cyst burden
		Riwanto et al. (2016)	Han:SPRD rat	↓cyst burden, ↑kidney function
		Chiaravalli et al. (2016)	^a^ *Pkd1* ^*ΔC/flox*^ *TmCre* mouse (inactivation of *Pkd1* at two time points)	↓cyst burden (both medium- and long-term models), ↑kidney function and ↓inflammation (long-term model)
		Lian et al. (2019)	^a^Inducible deletion of PKD1 in minipig	↓cyst burden, ↑kidney function
Metformin	AMPK agonist	Riwanto et al. (2016)	Human ADPKD cells	↓mRNA expression of glycolytic genes and ↓lactate
		Takiar et al. (2011)	• MDCK cells cultured with forskolin/IBMX^;^ • Embryonic C57/B6 mouse kidneys cultured with cAMP^;^ • ^a^ *Pkd1* ^*flox/-*^ *:Ksp-Cre* mouse^;^ • ^a^ *Pkd1* ^*flox/-*^ *:pCX-CreER* mouse	↓cyst size ↓cyst area ↓cyst burden ↓cyst burden
		Lian et al. (2019)	^a^Inducible deletion of PKD1 in minipig	↓cyst burden, ↑kidney function
		Chang et al. (2017)	^a^Morpholino knock-down of *pkd2* in zebrafish embryo	↓pronephric cyst burden and ↓inflammation
		Pastor-Soler et al. (2022)	^a^Homozygous R3277C point mutation in mouse *Pkd1*	↓cyst burden, ↑kidney function
Salsalate	AMPK agonist	Leonhard et al. (2019)	^a^iKsp-*Pkd1* ^del^ conditional knock-out mouse	↓cyst burden, ↑kidney function
Fenofibrate	PPARα agonist	Hajarnis et al. (2017)	^a^ *Pkd2* knock-out mouse	↓cyst burden
		Lakhia et al. (2018)	^a^ *Pkd1* ^*RC/RC*^ mouse	↓cyst burden, ↑kidney function, ↓inflammation

2DG: 2-deoxy-D-glucose; cAMP: cyclic AMP; IBMX: 3-isobutyl-1-methylxanthine; MDCK: Madin-Darby canine kidney.

a Orthologous animal model.

Interestingly, mTORC1 signaling also stimulates aerobic glycolysis, also known as the Warburg effect, by enhancing the translation of critical mediators such as c-Myc and hypoxia-inducible factor 1α (HIF-1α), which upregulate many glycolytic genes (Figure 1). (Osthus et al., 2000; Thomas et al., 2006; Lee et al., 2020) Aerobic glycolysis refers to the predominant cellular use of glycolysis to generate ATP, as opposed to the more metabolically efficient oxidative phosphorylation, even in the presence of abundant oxygen (Vander Heiden et al., 2009). Aerobic glycolysis is an important metabolic feature of proliferating cells (such as cancer cells) that facilitates replication of the cellular biomass (nucleotides, amino acids, and lipids) by providing specific molecular components (e.g., reduced nicotinamide adenine dinucleotide phosphate or NADPH) (Vander Heiden et al., 2009). Importantly, the Warburg effect is also a feature of cystic epithelia in ADPKD. Sirolimus reduced the expression of glycolytic genes in human ADPKD cells (Riwanto et al., 2016). Studies in rodents and minipigs showed that inhibiting glycolysis with 2-deoxy-D-glucose (2DG) improved cystic disease (Table 1) (Rowe et al., 2013; Rowe and Boletta, 2014; Chiaravalli et al., 2016; Riwanto et al., 2016; Lian et al., 2019).

However, in studies of patients with ADPKD, treatment with sirolimus and everolimus has produced disappointing results (Table 2). In randomized controlled trials of either sirolimus or everolimus, beneficial effects on TKV were short-lived or confounded by flaws in study design (e.g., small sample size or short follow-up) (Perico et al., 2010; Serra et al., 2010; Walz et al., 2010; Ruggenenti et al., 2016). In two of these studies, side effects from sirolimus prompted investigators to reduce the delivered doses below those intended, therefore potentially jeopardizing the effectiveness of the drug (Perico et al., 2010; Serra et al., 2010). A sirolimus trial including patients with advanced CKD was terminated after 1 year due to accelerated disease progression and multiple side effects (Ruggenenti et al., 2016). Lower-dose sirolimus might be a viable alternative, with evidence of fewer side effects (Braun et al., 2014). In addition, the study protocol for a randomized controlled trial of pulsed (weekly) oral sirolimus was published previously, although at this time the results remain unknown (*NCT02055079*) (Riegersperger et al., 2015). In conclusion, pharmacological inhibition of mTORC1 at present is not a promising clinical option for the treatment of ADPKD.

**TABLE 2:** Clinical studies investigating metabolic agents of interest in the treatment of ADPKD.

*Agent*	Mechanism of action	Study	Evidence	Advantages/Disadvantages of the therapy
Sirolimus	mTOR inhibitor	Serra et al. (2010) RCT	Inconclusive (low number of patients, short follow-up, no difference in TKV)	• Multiple side effects • Accelerated disease progression in patients with advanced CKD
		Perico et al. (2010) RCrT		
		Braun et al. (2014) RCT		
		Ruggenenti et al. (2016) RCT		
		Ongoing trial: NCT02055079 (status unknown)		
Everolimus	mTOR inhibitor	Walz et al. (2010) RCT	Inconclusive (effect on TKV not maintained)	• Multiple side effects
Metformin	AMPK agonist	Sorohan et al. (2019) SA	Good tolerability, studies not designed to assess change in kidney function	• Well-known, inexpensive drug• G-I side effects • Risk of lactic acidosis with lower kidney function
		Perrone et al. (2021) RCT		
		Brosnahan et al. (2022) RCT		
		Ongoing trials: NCT03764605 (status unknown); NCT04939935 (not yet recruiting)		

CKD: chronic kidney disease; RCT: randomized controlled trial; RCrT: randomized crossover trial; G-I: gastro-intestinal; SA: single-arm pilot study; TKV: total kidney volume.

### AMP-activated protein kinase is a potential therapeutic target in both obesity and autosomal dominant polycystic kidney disease

**FIGURE 2:**
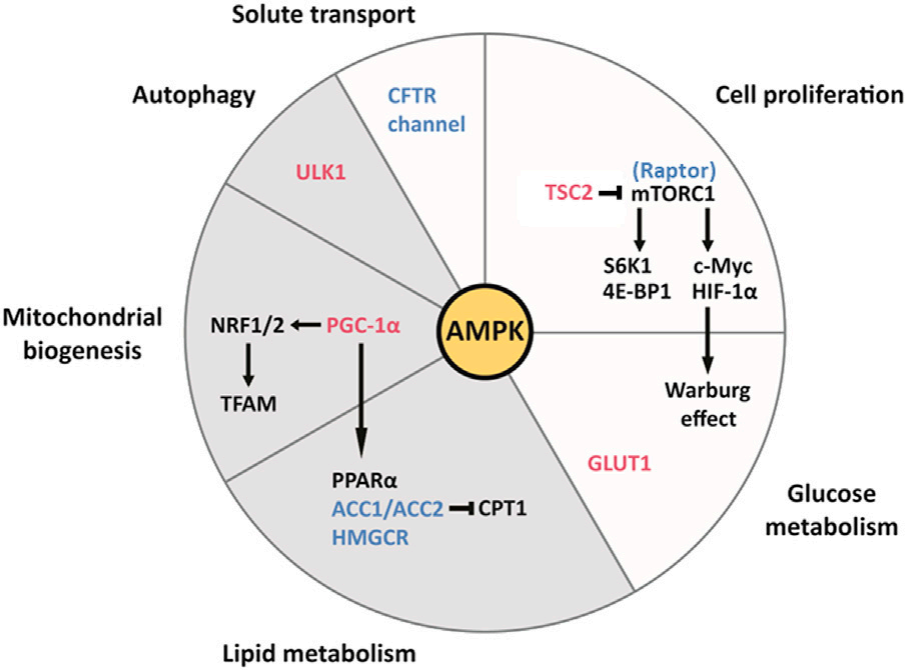
The pleiotropic effects of AMPK on cell function and metabolism in the kidney, and their relevance to ADPKD and obesity-related kidney disease. AMPK activates the downstream effectors colored in red and inhibits those colored in blue. Each slice of the pie chart summarizes pathways involved in the pathogenesis of ADPKD (white and grey) and lipid-induced nephrotoxicity seen with obesity (grey only). Clockwise from the top. AMPK inhibits mTORC1 by directly inhibiting the Raptor component of the complex and by activating TSC2, which inhibits mTORC1. mTORC1 is highly active in cyst cell linings and drives cellular proliferation through its downstream effectors S6K1 and 4E-BP1. By enhancing the translation of c-Myc and HIF-1α, mTORC1 contributes to the Warburg effect (aerobic glycolysis), which supports the replication of the cellular biomass necessary for proliferation. Additionally, AMPK enhances the translocation of the transmembrane transporter GLUT1 to the plasma membrane of baby hamster kidney (BHK) cells, which contributes to glucose uptake in response to insulin (Baldwin et al., 1997). AMPK inhibits ACC1/ACC2 and HMGCR, therefore suppressing fatty acid and cholesterol synthesis, respectively. ACC production of malonyl-CoA inhibits carnitine palmitoyl-transferase 1 (CPT1), which is required for the entry of fatty acyl-CoA into the mitochondria. Therefore, by inhibiting ACC, AMPK also stimulates fatty acid oxidation (Steinberg and Carling, 2019). Because of the intrinsic defect in fatty acid oxidation seen in ADPKD, which is associated with downregulation of the PPARα gene network, the intracellular accumulation of fatty acids may contribute to cystogenesis. Furthermore, lipid-induced nephrotoxicity appears to result from suppression of AMPK activity by fatty acid overload, leading to activation of ACC1 and HMGCR, and to suppression of PPARα expression. AMPK promotes mitochondrial biogenesis via PGC-1α, which activates NRF1 and NRF2, both of which regulate TFAM, a crucial transcription factor for mitochondrial DNA transcription and replication. By phosphorylating PGC-1α, AMPK activates PPARα. Defects in mitochondrial morphology and function are a hallmark of ADPKD. Furthermore, HFD can reduce the kidney expression of NRF1 and TFAM, thereby compromising mitochondrial biogenesis, in addition to inhibiting PGC-1α (Wang et al., 2018). AMPK phosphorylates ULK1, which initiates autophagy. The latter process is essential to maintain the homeostasis of renal tubular epithelial cells and appears to be defective in ADPKD. Lipid overload stimulates autophagy in the kidney proximal tubule and long-term lipid-induced autophagic activation can stress the lysosomal system, resulting in lysosomal dysfunction, impaired autophagy, and susceptibility to kidney injury. AMPK inhibits the CFTR channel responsible for intra-cystic fluid secretion.Figure adapted from Song et al. (2020)

AMPK, an important integrator of multiple pathways, is a therapeutic target of particular interest. AMPK functions as a major molecular sensor of cellular energy, responding to energy depletion by switching on catabolic pathways and switching off anabolic pathways (Hardie et al., 2012). As summarized in a recent review, AMPK plays a central role in the pathophysiology of ADPKD by modulating multiple biological processes, including cellular proliferation, autophagy, solute transport, metabolism and mitochondrial homeostasis in the kidney, all of which are important for cystogenesis (Figure 2). (Song et al., 2020)

AMPK is a heterotrimer consisting of a catalytic α-subunit, a regulatory β-subunit, and a regulatory γ-subunit assembled in a 1:1:1 ratio (Hallows et al., 2010; Hardie et al., 2012). Subunits have multiple isoforms (α1, α2, β1, β2, γ1, γ2, and γ3), each encoded by a different gene with tissue-specific expression and activity (Steinberg and Kemp, 2009). Isoform frequency in the kidney is species-dependent; for instance, the β1 subunit is present in 93, 96, and 64% of kidneys of C57BL/6J mice, Wister Han rats, and humans, respectively (Fraser et al., 2005; Salatto et al., 2017). AMPK is activated by rising concentrations of AMP relative to ATP. Upon binding to AMP, AMPK is phosphorylated in its catalytic loop (at residue Thr172) by one of the three upstream AMPK kinases (Figure 1): liver kinase B1 (LKB1), Ca^2+^/calmodulin-activated protein kinase kinase β (CaMKKβ), or Tak1 kinase (TAK1) (Steinberg and Kemp, 2009; Hallows et al., 2010; Hardie et al., 2012).

**FIGURE 3:**
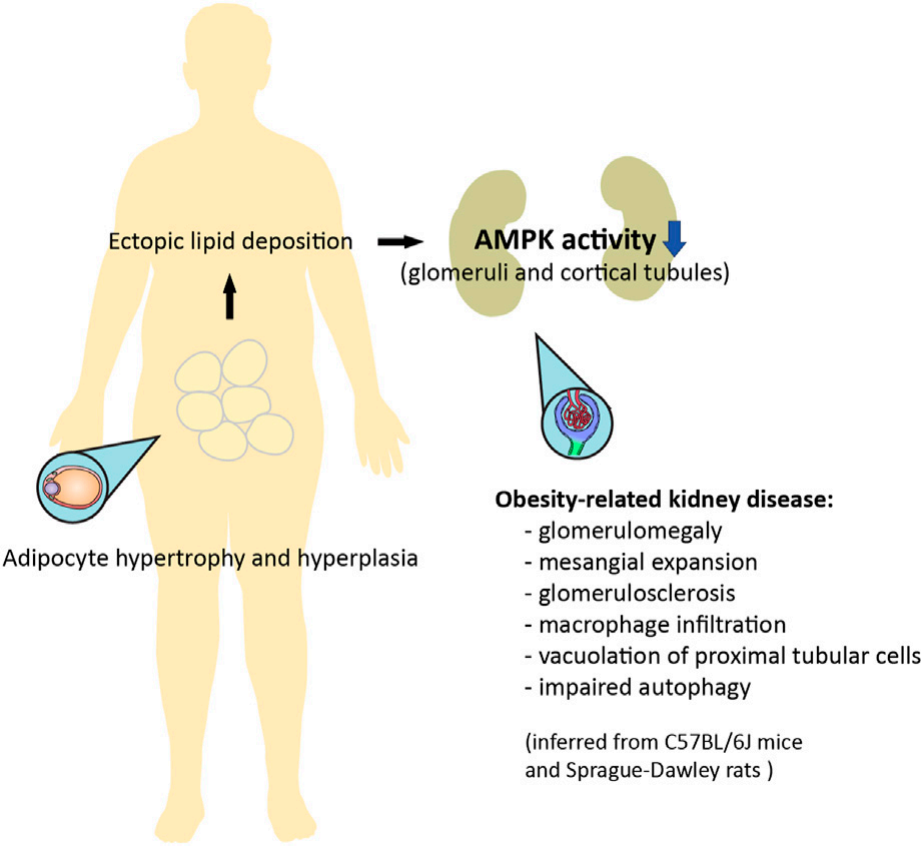
Ectopic lipid deposition in obesity can result in nephrotoxicity mediated by downregulation of AMPK activity. Adipocyte hypertrophy and hyperplasia is associated with an increase in circulating lipids, which leads to ectopic lipid deposition. Obesity-related kidney disease, as inferred from rodent models, is mediated by inhibited AMPK activity in the glomeruli and cortical tubular epithelium, and is characterized by glomerular enlargement, mesangial expansion, glomerulosclerosis, inflammation, evidence of tubular damage, and impaired autophagy (Declèves et al., 2011; Declèves et al., 2014; Wang et al., 2018). Some of these features can be improved with pharmacological activation of AMPK.

It has been suggested that AMPK signaling may be suppressed with obesity (Figure 3) (Steinberg and Kemp, 2009). AMPK activity is reduced in the heart, liver, and skeletal muscle in genetic models of rodent obesity (Barnes et al., 2002; Yu et al., 2004; Wangyun and Unger, 2005; Sriwijitkamol et al., 2006; Steinberg et al., 2006). On the other hand, AMPK activity is not always altered in the tissues of animals with HFD-induced obesity, nor is it altered in the skeletal muscle of obese humans (Steinberg et al., 2004; Bandyopadhyay et al., 2006; Martin et al., 2006). Nevertheless, AMPK activity is strongly downregulated in the adipose tissue of obese humans (Galic et al., 2011). This inhibition of AMPK may be related to low-grade chronic inflammation and high levels of glucose and insulin, which collectively suppress kinase activity through several distinct mechanisms (Steinberg et al., 2006; Valentine et al., 2014; Zhao et al., 2018; Jiang et al., 2021). With respect to lipid metabolism, AMPK inhibits acetyl-CoA carboxylase 1 and 2 (ACC1 and ACC2), thereby increasing fatty acid oxidation and suppressing fatty acid synthesis; AMPK also inhibits hydroxy-3-methylglutaryl-CoA reductase (HMGCR), therefore suppressing cholesterol synthesis (Steinberg and Kemp, 2009; Steinberg and Carling, 2019). This may be of special importance to the pathogenesis of lipid-induced nephrotoxicity, a type of ectopic lipid accumulation associated with obesity (Figures 2, 3) (van Herpen and Schrauwen-Hinderling, 2008). Indeed, AMPK was demonstrated to be an important pathway in HFD-associated kidney disease, described by the authors as glomerulomegaly with increased mesangial matrix, inflammation, and albuminuria. Specifically, HFD feeding in mice markedly decreased phosphorylation of glomerular AMPKα. Treating C57BL/6J mice with the AMPK activator 5-aminoimidazole-4-carboxamide ribonucleoside (AICAR) for 1 week restored AMPK activity, preventing kidney hypertrophy and macrophage infiltration (Declèves et al., 2011). However, it should be noted that AICAR has a number of AMPK-independent effects, and can lower blood pressure and blood glucose; as such, these results should be interpreted with caution. Interestingly, in a later study, HFD-induced renal injury was further described as vacuolated proximal tubular cells with loss of the brush border, suggesting tubular damage (Declèves et al., 2014). Glucagon-like peptide-1 (GLP-1) receptor agonist liraglutide was shown to restore AMPK signaling, and reduce glomerular size, glomerulosclerosis, and interleukin levels in obese Sprague-Dawley rats fed a HFD (Wang et al., 2018). Therefore, obesity-related kidney disease associated with lipid deposits appears to be mediated by suppressed AMPK activity, affecting both the glomerular and tubular compartments of the kidney.

Following the initial preclinical research examining the role of mTORC1 in ADPKD, several studies focused on its inhibitor AMPK. More than 10 years ago, metformin, an AMPK agonist, was used to suppress cystogenesis in an *in vitro* model, an *ex vivo* model, and two mouse models of ADPKD (Table 1). Metformin was shown to reduce mTOR signaling in the kidneys of the two mouse models (Takiar et al., 2011). The authors concluded that AMPK activation may alleviate cystic disease by two main mechanisms: by inhibiting the pro-proliferative mTOR pathway and the cystic fibrosis transmembrane conductance regulator (CFTR) channel that mediates fluid secretion into cysts (Takiar et al., 2011). In a subsequent study, AMPK phosphorylation was demonstrated to be reduced in fibroblasts isolated from *Pkd1* knock-out mouse embryos compared to wild-type cells (Rowe et al., 2013). In addition to its inhibitory effect on mTORC1, AMPK may potentially suppress cell proliferation in cysts by phosphorylating and activating eukaryotic elongation factor 2 kinase (eEF2K), which plays an essential role in inhibiting protein synthesis (Steinberg and Kemp, 2009). The distribution of AMPK in the kidney further supports its role in cystogenesis, as phosphorylated AMPK was detected on the basolateral surface of the rat collecting duct, which in the adult kidney is a major site of cyst formation (Fraser et al., 2005; Harris and Torres, 2009). While the molecular cause for suppressed AMPK activity in ADPKD remains unclear, *Pkd1* knock-out mouse embryonic fibroblasts (MEFs) displayed increased activity of the extracellular signal-regulated kinase (ERK). In the same experiment, ERK inhibited TSC2, which may contribute to mTORC1-mediated cell proliferation (Distefano et al., 2009). Although ERK has been postulated to suppress the LKB1-AMPK axis in *Pkd1* knock-out MEFs, this pathway remains to be confirmed (Rowe et al., 2013).

### Pharmacological agonists of AMP-activated protein kinase show promise in the treatment of autosomal dominant polycystic kidney disease

Several drugs that activate AMPK may have applications in the treatment of APDKD. Metformin, an oral agent commonly used to treat type 2 diabetes, activates AMPK indirectly by inhibiting complex I of the electron transport chain, leading to a drop in cellular ATP and an increase in AMP (Rena et al., 2017). In an *in vitro* model of kidney cystogenesis, metformin inhibited the expression of glycolytic genes and lowered lactate production (Riwanto et al., 2016). Several animal studies have demonstrated that metformin improves cystic disease (Table 1). In two *Pkd1* knock-out mouse models, metformin ameliorated cystic kidney disease (Takiar et al., 2011). Moreover, in MEFs isolated from *Pkd1* knock-out embryos, phosphorylated AMPK was reduced compared to wild-type cells; treating the mutant cells with metformin or AICAR restored AMPK activity (Rowe et al., 2013). In a minipig model and a zebrafish model, metformin similarly activated AMPK, thereby reducing cyst formation (Chang et al., 2017; Lian et al., 2017; Lian et al., 2019). Importantly, metformin improved disease severity in the *Pkd1* RC/RC mouse model, which approximates the slow progression of cystic disease in humans (Pastor-Soler et al., 2022). Pilot metformin studies in human populations with ADPKD did not show a benefit on eGFR, although the drug was well tolerated in both a single-arm study and two randomized controlled trials (Table 2). (Sorohan et al., 2019; Perrone et al., 2021; Brosnahan et al., 2022) Two additional trials of metformin in ADPKD are ongoing in Italy and Australia (*NCT03764605* and *NCT04939935*, respectively).

Canagliflozin, an SGLT2 inhibitor used to treat type 2 diabetes, has the off-target effect of activating AMPK indirectly by inhibiting mitochondrial respiration (Leonhard et al., 2019). By contrast, salicylate derived from the pro-drug salsalate activates AMPK directly by binding to its β-subunit. Salsalate, unlike aspirin, interacts little with the cyclooxygenase pathway, which limits the occurrence of gastrointestinal and bleeding side effects (Anderson et al., 2014). In a collaboration between our group and Leiden University, salsalate alone or in combination with metformin was shown to decrease kidney weight and improve survival from ESKD in an adult-onset conditional *Pkd1* knock-out mouse model. Conversely, treatment was ineffective with metformin alone, canagliflozin alone, or metformin in combination with canagliflozin (Leonhard et al., 2019). In this study, the method of administration of metformin was different (oral) from that used by Takiar et al. (2011) (intra-peritoneal), but the dosage was identical; the relatively low oral bioavailability of metformin (∼30–60%) likely limited its action in the kidneys (Padwal et al., 2011). Although the same oral metformin dosage was used in the *Pkd1* RC/RC mouse study, the longer duration of treatment in the latter may explain the improvement seen in kidney disease (Pastor-Soler et al., 2022). To our knowledge, there are currently no clinical trials of salsalate in ADPKD.

Lastly, AMPK-activating resveratrol, a polyphenol that also activates sirtuin 1 (SIRT1), was used to reverse diabetic nephropathy and prevent lipid-induced nephrotoxicity in db/db mice (Kim et al., 2013). Nonetheless, because treatment with a pan-sirtuin inhibitor (nicotinamide) and a SIRT1-specific inhibitor (EX-527) delayed cyst growth in *Pkd1* knock-out mouse embryonic kidneys, *Pkd1* conditional knock-out postnatal mice, and *Pkd1* hypomorphic mice, it is unclear whether sirtuin 1 is an interesting drug target in combined obesity and ADPKD (Zhou et al., 2013). In conclusion, AMPK-activating drugs such as metformin and salsalate are clinically easier to tolerate than mTORC1 inhibitors, making them more promising candidates to control cystogenesis in ADPKD. However, more clinical trials are needed.

### AMP-activated protein kinase interacts with other processes relevant to the pathophysiology of autosomal dominant polycystic kidney disease

In addition to modulating cell proliferation and fluid secretion, AMPK regulates other processes that have been shown to be abnormal in ADPKD, such as mitochondrial structure and function, the peroxisome proliferator-activated receptor α (PPARα) pathway, and the innate immune response (Figure 2). (Song et al., 2020)

#### The kidney displays mitochondrial abnormalities in both obesity and autosomal dominant polycystic kidney disease

Strong evidence indicates that AMPK promotes mitochondrial biogenesis by activating peroxisome proliferator-activated receptor γ coactivator-1 α (PGC-1α) (Steinberg and Kemp, 2009). As a transcriptional coactivator, PGC1α interacts with nuclear respiratory factors 1 and 2 (NRF1 and NRF2), which regulate mitochondrial transcription factor A (TFAM), the latter playing a crucial role in maintaining mitochondrial DNA transcription and replication. (Figure 2). (Kelly and Scarpula, 2004; Reznick and Shulman, 2006)

Mitochondrial dysfunction, presumably related to insulin resistance, is present in obesity, affecting tissues that participate in nutrient metabolism such as the adipose tissue, liver, and skeletal muscle (Bournat and Brown, 2010; Montgomery and Turner, 2015). The progressive enlargement of white adipose tissue in mice and humans can impair blood flow and result in hypoxia, which can lead to macrophage recruitment and infiltration (Wood et al., 2009). In turn, inflammation, in addition to excessive food intake, may increase the production of reactive oxygen species, causing mitochondrial dysfunction, although there is also evidence the latter may in fact precede adipose tissue inflammation (Lahera et al., 2017; de Mello et al., 2018; Woo et al., 2019). Increased mitochondrial fission, defective mitochondrial biogenesis and oxidative capacity, and intracellular triglyceride overload have been described in the adipose tissue, liver, and skeletal muscle of obese rodents and humans (Bournat and Brown, 2010; de Mello et al., 2018). In the kidney specifically, mitochondrial abnormalities have been inconsistently described in *ob/ob* mice and mice with HFD-induced obesity (Declèves et al., 2014; Szeto et al., 2016). Liraglutide restored AMPK and PGC-1α signaling in the kidneys of HFD-fed rats, and inhibited the formation of mitochondrial reactive oxygen species (Wang et al., 2018). There is also evidence that SS-31, an antioxidant that protects the structure of mitochondrial cristae, may prevent the renal toxicity, including mitochondrial damage, caused by fatty acid accumulation (Szeto et al., 2016).

The role of mitochondria in the pathophysiology of ADPKD has been covered in depth in several recent reviews (Padovano et al., 2018; Menezes and Germino, 2019; Nowak and Hopp, 2020; Podrini et al., 2020). Briefly, mitochondrial abnormalities can be found in *Pkd1* knock-out kidney epithelial cells isolated from mice, in mouse and rat models of polycystic kidney disease, and in kidney tissues from patients with ADPKD (Ishimoto et al., 2017; Lin et al., 2018). Morphological changes on electron microscopy include swelling and fragmentation of the mitochondria, and damaged cristae. These morphological changes in mitochondria, suggestive of defects in mitophagy, are very analogous to those observed in mice deficient for AMPK in muscle and brown adipose tissue (Bujak et al., 2015; Mottillo et al., 2016). Moreover, the mitochondrial DNA copy number decreases from an early stage of ADPKD, in parallel with kidney mRNA and protein expression of PGC-1α (Ishimoto et al., 2017). Several studies have highlighted that the impairment of mitochondrial metabolic processes such as fatty acid oxidation and oxidative phosphorylation drives cyst formation, in part *via* reduced PPARα expression (Menezes et al., 2012; Rowe et al., 2013; Menezes et al., 2016; Hajarnis et al., 2017; Padovano et al., 2017; Podrini et al., 2018). Because of defective fatty acid oxidation, a higher fat intake aggravated the kidney cyst burden in a non-orthologous rat and two orthologous mouse models of polycystic kidney disease (Jayapalan et al., 2000; Menezes et al., 2016). Since PC1 and PC2 appear to affect both the morphology and function of mitochondria, mitochondrial dysfunction might be one of the original molecular events preceding metabolic dysregulation in ADPKD (Padovano et al., 2017; Lin et al., 2018; Kuo et al., 2019).

#### Peroxisome proliferator-activated receptor α activation ameliorates lipid-induced toxicity in the kidney

AMPK phosphorylates, thereby activating, PGC-1α, which acts as a transcriptional coactivator of PPARα (Figure 2). (Vega et al., 2000; Steinberg and Kemp, 2009) The AMPKα subunit can also coactivate PPARα independently of its kinase activity (Bronner et al., 2004). This ligand-activated transcription factor binds to fatty acids to regulate essential metabolic responses to fasting, including ketogenesis in the liver and fatty acid utilization in multiple tissues such as the kidney (Kersten et al., 1999; Guan, 2002; Wang, 2010). In a mouse model of HFD-induced glomerular injury, treatment with the PPARα agonist fenofibrate increased the expression of lipolytic enzymes, reducing lipid accumulation and oxidative stress in the glomeruli, and preventing glomerular fibrosis (Tanaka et al., 2011). The network comprising PPARα and its gene targets is known to be downregulated in mouse models of ADPKD and human cysts, and PPARα downregulation is in part responsible for the intrinsic defect in fatty acid oxidation seen in ADPKD (Hajarnis et al., 2017; Lakhia et al., 2018; Lakhia, 2020). One mechanism underlying PPARα downregulation may be post-transcriptional inhibition by the microRNAs miR-17–92 and miR-21, which are upregulated in mouse and human ADPKD (Lakhia et al., 2016; Hajarnis et al., 2017). Using fenofibrate improved the phenotype of both aggressive and slowly progressive mouse models of ADPKD, and ameliorated the tubulointerstitial fibrosis associated with reduced fatty acid oxidation (Table 1). (Kang et al., 2015; Hajarnis et al., 2017; Lakhia et al., 2018) However, although fibrates are commonly used in the treatment of dyslipidemia, their long-term safety in kidney disease remains unclear (Jun et al., 2012; Melissa et al., 2020).

#### Chronic inflammation is a feature of obesity and autosomal dominant polycystic kidney disease

Both obesity and ADPKD are associated with a state of low-grade chronic inflammation. While other immune components participate in the inflammatory phenotype in both conditions, macrophages have been most extensively studied and play a dominant role (Zimmerman et al., 2020). In obesity, the upregulation of monocyte chemoattractant protein 1 (MCP-1) in fat depots with resulting accumulation of macrophages is crucial to induce inflammation in the adipose tissue (Oh et al., 2012; Amano et al., 2014). In humans, increased circulating MCP-1 correlates with higher levels of other inflammatory markers such as C-reactive protein (CRP) and interleukin-6 (IL-6), but also obesity, waist circumference, and the homeostatic model assessment for insulin resistance (HOMA-IR) score (Kim et al., 2006). Studies in obese mice support the causal role for MCP-1-mediated macrophage accumulation in adipose tissue in promoting insulin resistance (Kamei et al., 2006; Kanda et al., 2006; Weisberg et al., 2006; Aouadi et al., 2013; Kim et al., 2016). Interestingly, macrophage AMPKβ1 appears to blunt the inflammatory response to saturated fatty acids by increasing fatty acid oxidation (Galic et al., 2011).

In patients with ADPKD, the inflammatory markers MCP-1 and macrophage migration inhibitory factor (MIF) are elevated in the urine and cyst fluid (Zheng et al., 2003; Meijer et al., 2010; Kim and Tam, 2011; Chen et al., 2015). Urinary MCP-1 correlates with TKV and predicts progression to CKD stage 3 (Meijer et al., 2010; Chapman et al., 2012; Messchendorp et al., 2018; Segarra-Medrano et al., 2020). In an inducible knock-out model of *Pkd1*, MCP-1 upregulation, independently of tubular injury, caused monocyte infiltration. Monocytes differentiated into pro-inflammatory M1-like macrophages that accumulated around cysts, causing oxidative damage and tubular injury; a switch to alternatively activated M2-like macrophages then coincided with increased cyst proliferation (Cassini et al., 2018). In fact, most interstitial macrophages in the kidneys of patients with ADPKD were found to be M2-like, and human cyst cells were shown to promote M2-like macrophage polarization *in vitro* by secreting soluble factors including lactates (Swenson-Fields et al., 2013; Yang et al., 2018). M2-like macrophages, normally involved in tissue repair, are likely locked in a maladaptive cycle of pro-proliferative and pro-fibrotic activity (Weimbs, 2018). Previous work reported that global macrophage depletion with liposomal clodronate slowed kidney disease progression in non-orthologous and orthologous mice (Karihaloo et al., 2011; Swenson-Fields et al., 2013; Yang et al., 2018). Moreover, attenuating macrophage accumulation in *Pkd1*-deficient mice with *Mcp1* knock-out or an antagonist of the MCP-1 receptor (also known as CCR2) reduced cyst growth and tubular injury (Cassini et al., 2018; Viau et al., 2018).

Importantly, the proinflammatory cytokine MIF is associated on the one hand with increases in MCP-1, tumor necrosis factor α (TNF-α), and macrophage accumulation, and on the other hand with increased glucose uptake and glycolytic gene expression, inhibition of AMPK activity, and activation of ERK and mTOR signaling (Chen et al., 2015). Its deletion or pharmacologic inhibition has reversed these abnormalities and slowed disease progression in multiple *Pkd1*-deficient murine models (Chen et al., 2015). As an additional link between kidney inflammation and metabolic disease, LKB1 forms a complex with PC1 to suppress the expression of MCP-1 in tubular epithelia; the tubular deletion of *Lkb1* or *Pkd1* restores MCP-1 expression and causes monocyte/CCR2+ macrophage recruitment (Viau et al., 2018). This is notable because ciliary LKB1 colocalizes with and activates AMPK (Boehlke et al., 2010; Mick et al., 2015). Thus, similarly to MIF, LKB1 appears to be an upstream regulator of both inflammatory and metabolic changes in ADPKD.

## Conclusion

The pathobiology of ADPKD is strongly linked to metabolic dysregulation, which may be secondary to mitochondrial abnormalities induced by defective polycystin function. Pathways common to both obesity-related kidney disease and ADPKD suggest that obesity with ectopic lipid deposition in the kidneys may contribute to the progression of cystic disease through various mechanisms, notably through inhibition of AMPK activity. Overweight and obesity are frequent in patients with ADPKD, and weight loss should be beneficial for both conditions (Pickel et al., 1093; Nowak et al., 2018). Further work will be needed to understand if and how weight loss can attenuate cystogenesis on a molecular level. In addition to dietary changes, pharmacological activation of AMPK may attenuate lipid-related kidney injury as well as the cystic burden. The repurposing of metabolic drugs to treat patients with ADPKD has seen limited development thus far. Nevertheless, based on promising *in vivo* data, it is reasonable to believe that well-known and relatively safe pharmacological agents such as metformin or salsalate, both of which activate AMPK, could attenuate lipid-induced nephrotoxicity and reduce cystic disease severity. Given the slow progression of human ADPKD, using pharmacological agents that are safe and tolerable will be critical.
